# Characterization of Physicochemical, Phenolic, and Volatile Profiles of Peach Wine Fermented by Different *Saccharomyces* and Non-*Saccharomyces* Yeast Strains

**DOI:** 10.3390/foods15010056

**Published:** 2025-12-24

**Authors:** Xiaoqing Zhang, Zhenzhen Lv, Wenbo Yang, Hui Liu, Qiang Zhang, Jiechao Liu, Zhonggao Jiao

**Affiliations:** 1 Zhengzhou Fruit Research Institute, Chinese Academy of Agricultural Sciences, Zhengzhou 450009, China; fruitcaas@163.com (X.Z.); lvzhenzhen@caas.cn (Z.L.); yangwenbo@caas.cn (W.Y.); liuhui@caas.cn (H.L.); zhangqiang02@caas.cn (Q.Z.); jiaozhonggao@caas.cn (Z.J.); 2 Zhongyuan Research Center, Chinese Academy of Agricultural Science, Xinxiang 453000, China

**Keywords:** peach wine, yeast, non-*Saccharomyces*, *Saccharomyces*, phenolic compound, volatile organic compound

## Abstract

Screening of suitable yeast strains is essential for high-quality fruit wine production. In this study, twelve *Saccharomyces* and non-*Saccharomyces* yeasts were evaluated for their performance in fermenting peach wines. Results showed that all strains completed alcoholic fermentation and produced ethanol levels within the typical range for fruit wines. *Saccharomyces* strains had higher ethanol production ability. Non-*Saccharomyces* yeast-fermented peach wines showed higher sugar-free extract and acidity. Fermentation by different yeast strains resulted in diverse characteristics of phenolic and volatile profiles in peach wines. The peach wine fermented by *S. cerevisiae* strain EC1118 was characterized by improved color parameters and higher antioxidant capacity. The non-*Saccharomyces* yeasts tended to produce more esters than alcohols. The *Saccharomyces* strains favored the production of alcohols more than esters. *P. fermentans* 33372 yielded a higher level of ethyl esters. *I. orientalis* 31129 produced higher levels of isoeugenol, linalool, and β-damascenone. Overall, non-Saccharomyces yeast strains appeared more promising for use on their own or in co-fermentation with *S. cerevisiae* strains to produce peach wines with a higher level of volatile organic compounds.

## 1. Introduction

Peaches [*Prunus persica* (L.) Batsch] are one of the most widely cultivated and commonly consumed fruits worldwide. In 2023, the global total cultivated area and production quantity of peaches reached 1,561,641 ha and 27,077,873 tons, respectively (from FAOSTAT data). Peaches are highly appreciated by consumers due to their richness in nutrients and bioactive phytochemicals [1]. However, the fresh peach is highly perishable as a climacteric fruit. It undergoes rapid postharvest softening and senescence, which may result in reduced marketability and great postharvest losses in some cases [2,3]. Therefore, it is essential to process fresh peaches into various products to expand the use of peaches, and thereby reduce the postharvest losses.

Winemaking is one of the most ancient and commercially prosperous biotechnological processes for fruit processing. By fermentation, the fruits were preserved by alcohol production, and the nutrition and flavor profiles of fruits were altered with the generation of distinctive nutritive and flavor characteristics [4]. Traditionally, wine is mainly produced from grapes. However, there is a trend to utilize various other fruits for producing wines with distinctive nutritive and sensory properties. The production of various fruit wines has been considered as an attractive approach for utilizing surplus fruits and reducing postharvest losses [4].

Yeast is the main microorganism employed in fruit wine production. The selection of suitable yeast strains is one of the most important procedures for high-quality fruit wine production [5]. Different yeast strains may exhibit diverse fermentation performances and metabolic responses in the fruit matrix, resulting in differences in characteristics for fruit wines [6,7,8,9]. Furthermore, the fermentation performance and metabolism of yeast may be influenced by the nature of different fruit matrices, showing a substrate-dependent manner [10]. *Saccharomyces* genus yeasts, predominantly *S. cerevisiae* strains, are the most frequent choice for their reliable performances for fermentation. *S. cerevisiae* is able to overcome almost all fermentation stresses during winemaking, such as acidic conditions, osmotic conditions, sulfur dioxide and ethanol toxicity, oxygen limitation, and temperature elevation [11]. Recently, non-*Saccharomyces* yeasts have gained increasing attention in modern winemaking because they can improve the flavor complexity and quality attributes of fruit wines [12,13,14,15]. Several studies have reported the application of non-*Saccharomyces* yeasts as an alternative or complement to *S*. *cerevisiae* in the fermentation of a variety of fruit wines, including passion fruit [8], kiwifruit [16], citrus [17], strawberry [18], pineapple [19], etc.

Peach wine production has developed significantly with the rapid increase in peach production in China. However, knowledge on yeast selection in terms of improving peach wine quality remains limited. Liu et al. [20] investigated the alcoholic fermentation performance of one *S*. *cerevisiae* strain and five non-*Saccharomyces* strains as well as the effects on and aroma profile of yellow-fleshed peach wine. It was suggested that a mixed culture of *S*. *cerevisiae* and non-*Saccharomyces* yeast could improve the quality of yellow-fleshed peach wine. However, the fermentation was conducted with concentrated yellow-fleshed peach juice. Generally, the concentrated peach juice has been subjected to many processing steps such as thermal treatment and vacuum concentration, thus resulting in great changes in physicochemical properties and chemical compositions [21]. Considering that the current fermentation of peach wine is more commonly performed with fresh-squeezed peach juice or puree, it is still necessary to evaluate the suitability and effects of more *Saccharomyces* and non-*Saccharomyces* yeast strains on fresh-squeezed peach juice or puree fermentation. Moreover, the peaches can be generally classified into three groups according to the flesh color, namely white-fleshed, yellow-fleshed, and red-fleshed. Among these groups, the white-fleshed peach is the most widely cultivated in China. Therefore, this work collected five *S*. *cerevisiae* strains, one *S. bayanus* strain, and six non-*Saccharomyces* yeast strains to ferment fresh-squeezed peach puree prepared from a widely cultivated white-fleshed peach variety ‘Chunmi’, and their effects on the physiochemical properties, phenolic composition, and volatile profile of peach wine were investigated. The findings will provide valuable insights into selecting suitable yeast strains for producing peach wines with high quality or different characteristics and benefits, especially fermentation with fresh-squeezed peach puree or juice prepared from white-fleshed peaches.

## 2. Materials and Methods

### 2.1. Materials

#### 2.1.1. Peaches

Peaches of the white-fleshed variety ‘Chunmi’ were harvested at commercial maturity (70 days after full bloom) from an orchard in Xinxiang, Henan Province, China. Fresh peaches with uniform size, base color of the peel, and firmness, and free of mechanical injury, insects, and disease were selected and washed with distilled water, and then the residual water on the fruit surface was removed by flow air.

#### 2.1.2. Yeast Strains

*S. cerevisiae* (Sc) Lalvin EC1118 (EC1118) and 71B-1122 (Sc71B) were obtained from Lallemand Inc. (Montreal, Canada). *S. cerevisiae* 2 (Sc2) was preserved in our lab in Zhengzhou Fruit Research Institute, CAAS. *S. cerevisiae* 1796 (Sc1796), *S. cerevisiae* 1458 (Sc1458), *S. bayanus* 1465 (Sb1465), and *Schizosaccharomyces pombe* 1260 (Schp1260); *Candida krusei* 1273 (Ck1273); *Issatchenkia orientalis* 31129 (Io31129); *Hanseniaspora uvarum* 32337 (Hu32337); *Pichia fermentans* 33372 (Pf33372); and *Rhodotorula mucilaginosa* 33374 (Rm33374) were purchased from CICC (China Center of Industrial culture Collection, Beijing, China).

### 2.2. Methods

#### 2.2.1. Peach Wine Preparation

The fresh peaches were pitted and pureed with a lab-scale food processor (JYL-G12E, Joyoung, Jinan, China). The resulting peach puree had a total soluble solid content of 10.3 °Brix, total acidity of 3.0 g/L, total phenolic content of 38.9 mg/L, and pH value of 3.9. This peach puree was combined with 60 mg/L of sulfur dioxide in the form of K_2_S_2_O_5_ to suppress oxidation and the growth of unexpected microbes. Meanwhile, sugar was added to chaptalize the must to 22.0 °Brix. The fermentation was started by inoculating different yeast inoculums at a cell density of 1 *×* 10^6^ CFU/mL. Triplicate fermentations (1 kg peach pulp) were carried out in a 2.5 L glass bottle at 20 ± 1 °C. The end of fermentation was recognized when the residual sugar remained almost unchanged. After fermentation, the wines were filtered, supplemented with 60 mg/L sulfur dioxide, and then sealed in glass bottles and stored at 20 ± 1 °C in the dark for 30 d before further analysis. The peach wine was centrifuged at 11,325× *g* for 15 min, and the resulting supernatant was used for the following determinations.

#### 2.2.2. Determination of Color Parameters

The samples were firstly filtered with a 0.45 μm microfilter, and then the determination of color parameters was performed by using a SP62-162 colorimeter (X-rite, Grand Rapids, MI, USA), employing the CIELAB system to measure the L* (lightness), a* (green-redness), and b* (blue-yellowness). The C* (chroma) and h* (hue angle) were calculated using the following formula (Equations (1) and (2)):

(1) C* = (a*^2^ + b*^2^) ^1/2^

(2) h* = arctan(b*/a*)

According to the CIELAB system, a negative a* value refers to green chroma, and a positive a* value represents red hue; a negative b* value refers to blueness, and a positive b* value represents yellowness; and a hue angle of around 90° represents yellowness.

#### 2.2.3. General Chemical Analysis

The ethanol content of peach wine was determined according to the alcoholmeter method described in the National Food Safety Standard of People’s Republic of China (GB 5009.225-2023) [22]. The total acidity, sugar-free extract, and residual sugar of peach wine were determined following the methods of the National Standard of People’s Republic of China (GB/T 15038-2006) [23]. Total acidity was determined by neutralization titration with 0.1 mol/L NaOH and calculated as the tartaric acid equivalent [24]. Total sugar content was analyzed by using a direct titration method with Fehling’s reagents and calculated as the glucose equivalent [25].

#### 2.2.4. Higher Alcohols Analysis

The higher alcohols in peach wine were analyzed by using a gas chromatography (GC) method according to the National Standard of People’s Republic of China (GB/T 5009.48-2003) [26]. This method can determine the contents of n-propanol, isobutanol, and isopentanol with a limit of detection (LOD) of 0.2, 0.22, and 0.15 ng, respectively. The higher alcohols analysis was performed on a GC2010 plus system (Shimadzu, Kyoto, Japan) equipped with a flame ionization detector (FID). The higher alcohols were separated on a FAMEWAX capillary column (30 m × 0.25 mm × 0.25 μm). The temperature of the injector and detector were held at 230 °C and 220 °C, respectively. The carrier gas of N_2_ was set at a flow rate of 0.8 mL/min, and the split ratio was set at 50:1. The oven temperature program was as follows: an initial temperature of 35 °C was held for 2.5 min, then raised to 100 °C at 8 °C/min and held for 4 min, and then further increased to 150 °C at 10 °C/min. Standards of n-propanol, isobutanol, and isopentanol were used to identify and quantify the higher alcohols by using the external method. The concentrations of identified higher alcohols were calculated using the following equations obtained from the calibration curves (Equations (3)–(5)).

(3) n-Propanol concentration (mg/L) = 1,447,027x − 219.60   (R^2^ = 0.99987)

(4) Isobutanol concentration (mg/L) = 1,596,485x − 126.97   (R^2^ = 0.99992)

(5) Isopentanol concentration (mg/L) = 1,616,731x − 163.10   (R^2^ = 0.99995)

where x is the peak area.

#### 2.2.5. Determination of Total Phenolic Content and Antioxidant Capacity

The total phenolic content (TPC) in peach wine was measured by using the Folin–Ciocalteu method as described in our previous research [27], with gallic acid being used as the standard. The in vitro antioxidant capacity of peach wine was determined by using the DPPH (1,1-diphenyl-2-picrylhydrazyl) scavenging method described by Yang et al. [25], and the results were reported as µmol of TEAC (Trolox equivalent antioxidant capacity) per liter of peach wine.

#### 2.2.6. High-Performance Liquid Chromatography Analysis of Phenolic Profile

The phenolic profile of peach wine was determined by using a Waters e2695 high-performance liquid chromatography (HPLC) system coupled with a 2998 photodiode array detector (Waters Chromatography Div., Milford, MA, USA) according to the method described in our previous research [28,29]. A Waters Symmetry C18 column (4.6 × 250 mm, 5.0 µm) was used to separate the phenolic compounds at 35 °C. The eluting solvents were acetonitrile (eluent A) and 2 g/L of aqueous acetic acid solution (eluent B). The flow rate of the mobile phase was 1.0 mL/min, and the gradient program was as follows: 0 min, 5% A; 3 min, 6% A; 4 min, 7% A; 10.0 min, 7% A; 12 min, 8% A; 24 min, 10% A; 30 min, 15% A; 35 min, 15% A; 50 min, 37% A; 55 min, 45% A; 60 min, 100% A; 70 min, 100% A; and 75 min, 5% A. This method allows for the determination of protocatechuic acid, catechin, vanillic acid, chlorogenic acid, syringic acid, epicatechin, procyanidin C1 p-coumaric acid, cinnamic acid, and isorhamnetin with an LOD of 0.00034, 0.039, 0.050, 0.14, 0.077, 0.12, 0.13, 0.050, 0.091, and 0.015 mg/L, respectively.

#### 2.2.7. Gas Chromatography–Mass Spectrometry Analysis of Volatile Organic Compounds

The volatile organic compounds (VOCs) in peach wine samples were analyzed following the methods described by Wu et al. [29] with minor modifications. In brief, 5 mL of peach wine together with 2 g of NaCl were transferred to a headspace vial (25 mL). Subsequently, 20 μL of internal standard solution (2-octanol, 500 μg/mL) was added and the resulting mixture was equilibrated at 40 °C for 20 min under agitation. After that, an aged 50/30 μm DVB/CAR/PDMS fiber (Supelco, Bellefonte, PA, USA) was inserted into the vial and exposed to the headspace for 40 min. The fiber was then removed from the vial and inserted into the injection port of the gas chromatography–mass spectrometer (GC-MS) to allow thermal desorption at 250 °C for 8 min. The VOCs were separated on a DB-225MS capillary column (30 m × 0.25 mm × 0.25 μm) by using a 7890-5975C GC-MS system (Agilent, Santa Clara, CA, USA). Helium was used as the carrier gas, and the flow rate was set at 1 mL/min. The temperature of the column was maintained at 33 °C for 1 min, then increased to 78 °C at a rate of 3 °C/min and held for 2 min, further increased to 99 °C at a rate of 3 °C/min and held for 2 min, increased to 140 °C at 3 °C/min and held for 1 min, increased to 160 °C at 6 °C/min, and finally increased to 220 °C at 8 °C/min and held for 2 min. The mass spectra were acquired in electron impact (EI) mode at 70 eV with a scan range of 50–500 *m*/*z*. The VOCs were identified by matching the mass spectra with those in the mass spectral database of National Institute of Standard and Technology (NIST), with a minimum library similarity match of 800. The relative contents of the VOCs were calculated by comparing the peak areas with that of the internal standard, and the relative odor activity value (ROAV) was calculated by comparing the relative concentration of each VOC with the odor threshold according to previous studies.

#### 2.2.8. Statistical Analysis

Statistical analysis was performed by using GraphPad Prism 8 (GraphPad Software Inc., San Diego, CA, USA). Replicates represent the independent fermentations. Prior to analysis, the assumption of normality of variances was verified using the Kolmogorov–Smirnov test. Statistical differences among different fermentation samples were then examined by one-way analysis of variance (ANOVA), and the post hoc multiple comparisons were carried out using Tukey’s test. The two-tailed Pearson correlation method was used to conduct correlation analysis. Levels of *p* < 0.05 were considered significant.

## 3. Results and Discussion

### 3.1. Physicochemical Characteristics

#### 3.1.1. Color Parameters

Color is one of the first perceived sensory characteristic of fruit wines. It can greatly affects consumers’ acceptance owing to its important effects on wine appearance. As shown in Table 1, all of the peach wines had similar lightness (L*), suggesting that yeast strains could not affect the lightness of peach wine. However, the parameters a* and b* were very dependent on the yeast strains used for fermentation. All of the samples had a negative a* value ranging from −0.01 to −0.75, except Schp1260, indicating a faint greenness. Conversely, the Schp1260-fermented peach wine showed a positive a* value, indicating a red hue. As for b*, all samples showed a positive value ranging from 7.51 to 12.68, which means they all had a yellow chroma. The hue angle values (h*) ranging from 85.69° to 89.31° also represented a yellow hue. These parameters suggest that the color of the peach wines was yellow with faint greenness or redness, depending on the yeast strains used for fermentation.

The color of fruit wine primarily results from the pigments in the fermentation matrix, which may subsequently undergo extensive biochemical changes during fermentation and storage [10,30]. The peach fruit contains carotenoids, such as β-carotene and lutein, giving a yellow color for peach and its products [31]. Furthermore, the phenolics in peach must may undergo oxidation reactions to form a yellowish brown color, which is a commonly occurring browning reaction in fruit wine production [32]. Considering that white-fleshed peach fruit usually has low level of carotenoids and the carotenoids are susceptible to oxidative degradation during winemaking [33], it is suggested that the yellowness in peach wine was mainly ascribed to the browning reaction by oxidation of phenolic compounds during fermentation and subsequent storage.

Some yeast strains have been reported to be able to produce glutathione (GSH). It can react with the quinones generated by the enzymatic oxidation of phenols, thereby blocking the browning reactions during the winemaking process [34,35]. Furthermore, the yeasts and their cell wall components can absorb browning substrates and products in wine, and as a result, are commonly used as fining treatment to correct browning in wine [36,37]. This suggests that the yeasts as well as their autolysates might modify the browning degree of wine during storage. Some non-*Saccharomyces* yeast strains such as *Metschnikowia pulcherrima* have also been reported to be able to effectively consume the oxygen in grape must, thereby protecting it from browning [35]. However, these effects may be strain-dependent due to the diverse characteristics of various yeast strains. In the present study, the Ck1273-, EC1118-, Sc2-, Rm33374-, Io31129-, Pf33372-, and Hu32337-fermented peach wines showed a lower yellowness (b*) and higher greenness (negative a*), suggesting that these yeast strains could retain the natural color of white-fleshed peach pulp. On the other hand, Sc1796 and Sc71B yielded a higher yellowness (b*) and Schp1260 led to a higher redness (positive a*), suggesting that they could not retain the natural color. Up to now, knowledge on the role of various yeast strains on the browning of fruit wine is scarce. Further research should be done to elucidate the mechanism of these yeast strains on the protection of peach wine color.

#### 3.1.2. Ethanol, Total Acidity, Residual Sugar, and Sugar-Free Extract

**Figure 1:**
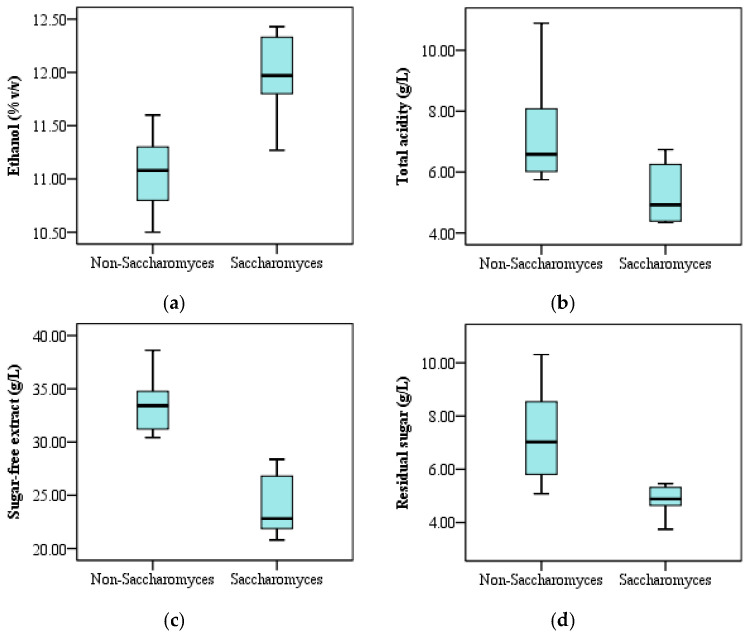
Comparison of ethanol (**a**), total acidity (**b**), sugar-free extract (**c**), and residual sugar (**d**) content of peach wine fermented by *Saccharomyces* and non-*Saccharomyces* yeast strains.

The predominant role of yeast in fruit wine fermentation is to metabolize sugars into ethanol. Thus, the ethanol production capacity usually serves as an important criteria for yeast selection. As shown in Table 2, the fermentation by different *Saccharomyces* and non-*Saccharomyces* yeast strains yielded an ethanol concentration ranging from 10.5% to 12.4% *v*/*v* in peach wines. This is within the typical range for fruit wines. Among the 12 yeast strains, EC1118 and Sb1465 showed the highest ethanol production capacities, whereas the peach wine fermented by Ck1273 was the lowest, with a 15.53% lower ethanol content than EC1118. It has been well recognized that *Saccharomyces* yeast strains generally possess relatively higher ethanol production capacity than non-*Saccharomyces* strains [20,38]. The present results also agree with this. The peach wines fermented by the *Saccharomyces* strains had a relatively higher ethanol concentration ranging from 11.27% to 12.43% *v*/*v*, while the ethanol content in peach wines fermented by the non-*Saccharomyces* yeast strains showed an ethanol content range of 10.50–11.60% *v*/*v* (Figure 1a). Notably, Rm33374, a non-*Saccharomyces* yeast strain, exhibited a comparable ethanol production capacity to EC1118 and Sb1465. Otherwise, Ck1273 and Hu32337 can be used for producing peach wine with a low alcohol level due to their lower ethanol production capacities. Nowadays, it has become a popular trend to produce fruit wines with a low alcohol level [39].

Organic acids play an important role in the flavor characteristics and chemical stability of fruit wines. As listed in Table 2, the total acidity of peach wine ranged from 4.34 g/L (Sc2) to 10.89 g/L (Schp1260). Significant difference in total acidity was observed in peach wines fermented by different yeast strains (*p* < 0.05). The non-*Saccharomyces* yeasts generally showed higher acidification ability than *Saccharomyces* strains (Figure 1b). The *S. pombe* strain 1260 had the highest acidification ability, yielding a total acidity of 10.89 g/L in peach wine. It has been reported that *Schizosaccharomyces pombe* produced more pyruvic acid and succinic acid during fermentation than *S. cerevisiae* [40,41], which might partly contribute to the higher level of total acidity in the resulting peach wine. A significant negative correlation was found between ethanol and total acidity (*p* < 0.05), indicating a reduction of carbon flow towards ethanol conversion.

The sugar-free extract in peach wines fermented by different yeast strains varied from 20.80 g/L (Sc2) to 38.61 g/L (Io31129), with a significant difference between *Saccharomyces* and non-*Saccharomyces* yeast strains (Table 2, Figure 1c). All of the non-*Saccharomyces* yeast strains’ fermentation yielded a sugar-free extract level higher than 30 g/L. However, the highest content of sugar-free extract for *Saccharomyces* strains was only 26.81 g/L. These results suggest that the non-*Saccharomyces* yeasts could facilitate the extraction of soluble substances from peach pulp. Villar et al. [42] also reported a higher content of sugar-free extract in rosé wine fermented by a non-*Saccharomyces* yeast than *S. cerevisiae*. This is similar to our results. There was a significant negative correlation between ethanol and sugar-free extract (*p* < 0.05), suggesting that the non-*Saccharomyces* yeasts might produce more soluble substances other than ethanol during fermentation.

The residual sugar in the peach wines fermented by different yeast strains ranged from 3.75 g/L (Sb1465) to 10.31 g/L (Hu32337), showing a significant negative correlation with ethanol (*p* < 0.01). The non-*Saccharomyces* yeasts exhibited a generally lower sugar consumption ability as compared with *Saccharomyces* strains (Figure 1d). This was in accordance with their ability to produce ethanol. The high level of residual sugar in Hu32337- and Ck1273-fermented peach wines might be one of the reasons for their low ethanol content.

#### 3.1.3. Higher Alcohols

**Figure 2:**
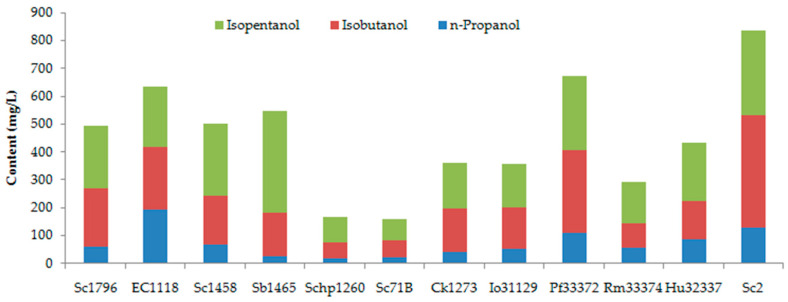
Higher alcohols in peach wines fermented by different yeast strains.

Three higher alcohols, namely n-propanol, isobutanol, and isopentanol, were identified in the peach wine. As illustrated in Figure 2, the peach wines fermented by different yeast strains showed an n-propanol level of 24.55–194.47 mg/L. The isobutanol concentration ranged from 57.83 to 401.53 mg/L, being 1.14–6.38 times higher than n-propanol. The isopentanol level was also higher than n-propanol, ranging from 77.90 to 364.80 mg/L. A significant difference was found among different yeast strains. The Schp1260- and Sc71B-fermented peach wine showed the lowest level of higher alcohols, with a total content of 167.33 mg/L and 160.28 mg/L, respectively. Sc2 fermentation yielded the highest content of isobutanol and total higher alcohols. EC1118-fermented peach wine had the highest level of n-propanol, with a total higher alcohol content of 634.82 mg/L. According to previous literature [43], higher alcohols at a level below 300 mg/L are commonly regarded to contribute a desirable complexity to the wine aroma. But at concentrations above 400 mg/L, they are usually regarded as playing an undesirable role in wine aroma. In the present study, only Rm33374, Io31129, and Ck1273 produced a moderate level of higher alcohols, with a total amount of 292.23, 355.24, and 359.04 mg/L, respectively. More than half of the peach wines had a total amount of higher alcohols exceeding 400 mg/L. Especially in the Sc2-, EC1118-, and Pf33372-fermented peach wines, the total higher alcohols exceeded 600 mg/L. These results suggest that some work needs to be done to reduce the higher alcohols in peach wines when using these yeast strains as starters for fermentation.

Higher alcohols are mainly formed by yeast during fermentation from amino acids in the fermentation matrix via the Ehrlich pathway or directly from sugars via the Harris pathway. Their formation can be affected by yeast strains and the nutritional composition of the must, as well as fermentation conditions [43,44,45]. Therefore, the regulation of the fermentation matrix and selection of yeast strains with low higher alcohols-producing ability are considered to be the primary approaches to reducing the level of higher alcohols in fruit wines. Furthermore, the higher alcohols in wine also can be reduced by microwave irradiation [46] and ultrasound irradiation [47]. Further research can also be carried out in terms of the reduction of higher alcohols in peach wines by regulating the fermentation process and applying novel technology to degrade higher alcohols in wine.

### 3.2. Phenolic Compounds and Antioxidant Capacity

#### 3.2.1. Total Phenolic Content and Antioxidant Capacity

**Figure 3:**
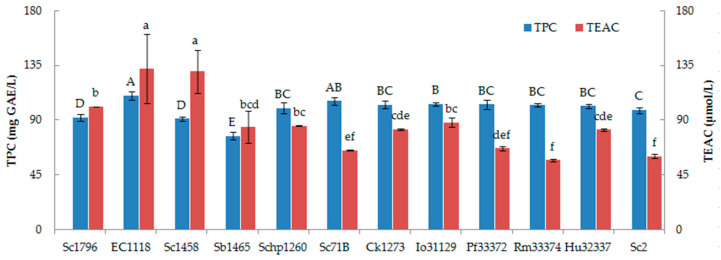
Total phenolic content and antioxidant capacity of peach wine fermented by different *Saccharomyces* and non-*Saccharomyces* yeasts. TPC: total phenolic content; TEAC: Trolox equivalent antioxidant capacity. The error bars represent standard deviation (SD). Different capital letters and lowercase letters indicate statistically significant differences (*p* ˂ 0.05) in TPC and TEAC, respectively.

The TPC in peach wines fermented by different yeast strains ranged from 77.11 to 110.44 mg GAE/L, with significant differences among different yeast strains (Figure 3). The highest TPC was observed in EC1118-fermented peach wine, whereas the Sb1465-fermented sample showed the lowest, being only 69.82% of EC1118. As for antioxidant capacity, the EC1118-fermented peach wine also showed the highest TEAC, while the Rm33374-fermented sample had the lowest TEAC. The antioxidant capacity of the peach wine was not always significantly correlated to TPC. This might be ascribed to the existence of other antioxidant compounds that are insensitive to the Folin–Ciocalteu reagent in peach wine.

#### 3.2.2. Phenolic Profile

**Figure 4:**
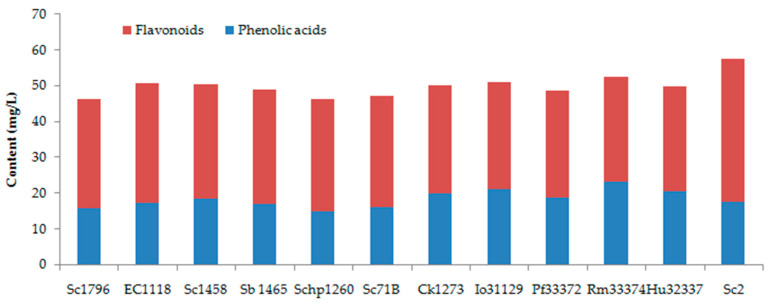
Phenolic composition of peach wines fermented by different yeast strains.

By using HPLC, a total number of ten phenolic compounds, including six phenolic acids and four flavonoids, were identified and quantified in the peach wines. As shown in Table 3, epicatechin was the most abundant phenolic compounds in the peach wines, contributing to 42.96–56.86% of the total amount of detected phenolic compounds. Accordingly, the total content of detected flavonoids was also higher than that of phenolic acids, accounting for 55.39–69.39% of the total content of detected phenolic compounds (Figure 4). Sc2 yielded the highest content of epicatechin and total flavonoids, while Rf33372-fermented peach wine had the most abundant phenolic acids. Procyanidin C1 and catechin were the second-most abundant flavonoids in peach wines, ranging from 2.45 mg/L to 5.04 mg/L and 2.87 mg/L to 3.67 mg/L, respectively. Protocatechuic acid, chlorogenic acid, syringic acid, and vanillic acid were the main phenolic acids in peach wines, with a content range of 2.42–8.64 mg/L, 5.52–6.58 mg/L, 4.87–6.27 mg/L, and 1.09–4.09 mg/L, respectively. EC1118-fermented peach wine had the highest content of procyanidin C1, which was 1.63–2.06 times of those in other samples. The protocatechuic acid in Rm33374-fermented peach wine was 1.03–3.57 times of those in other peach wines. The Sc2-fermented sample had a vanillic acid of 78.60–275.23% higher than other peach wines. There was no significant difference in each phenolic compound between *Saccharomyces* and non-*Saccharomyces* yeast strains except for protocatechuic acid, which showed a significant difference (*p* < 0.001) between these two groups of yeasts.

**Figure 5:**
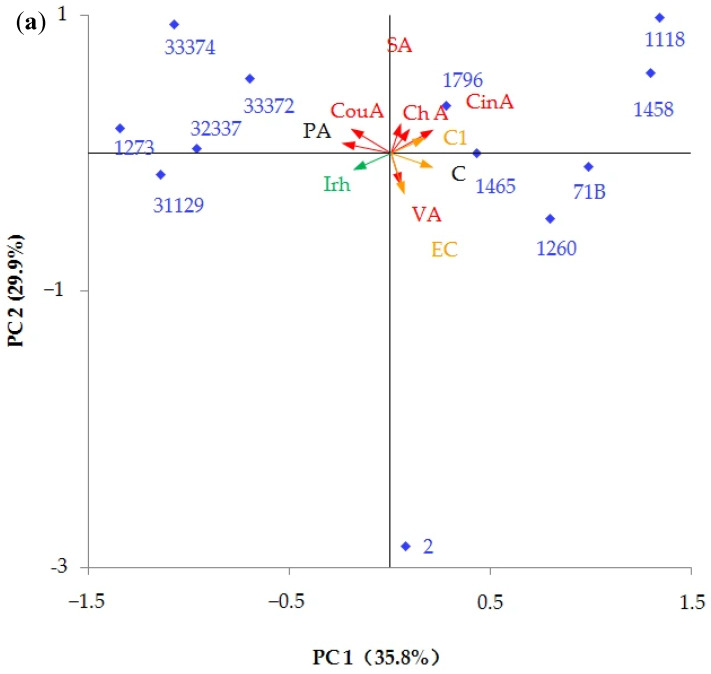

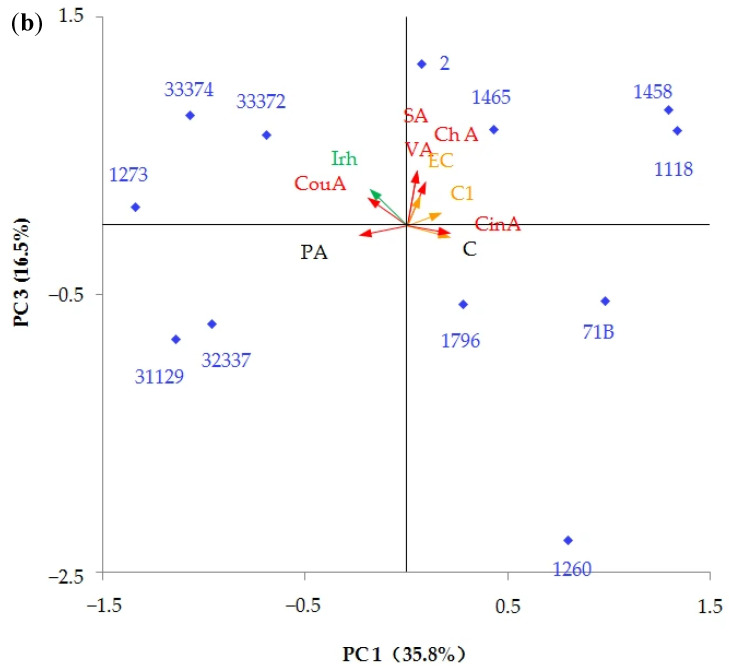

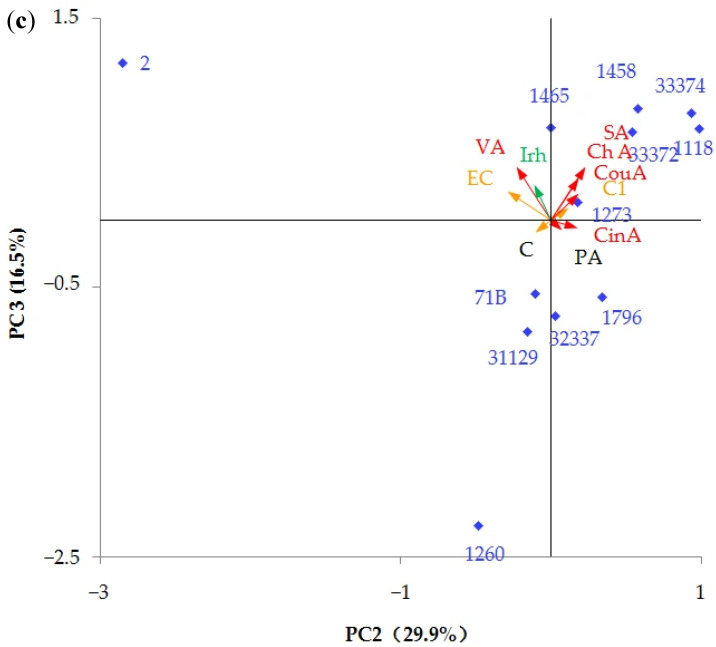
PCA score plots of phenolic compounds. (**a**) PC1 vs. PC2; (**b**) PC1 vs. PC3; and (**c**) PC2 vs. PC3. PA: protocatechuic acid; C: catechin; VA: vanillic acid; ChA: chlorogenic acid; SA: syringic acid; EC: epicatechin; C1: procyanidin C1; CouA: p-coumaric acid; CinA: cinnamic acid; Irh: isorhamnetin.

To better interpret the influence of different yeast strains on the phenolic profiles of peach wines, the data on the phenolic compounds in different samples were visualized by using a principal component analysis (PCA) map. Three principal components (PCs), which could explain 82.19% of the variance in the original data, were extracted for score plots. As demonstrated in Figure 5, most of the peach wines had a distinct characteristic phenolic profile from others. Sc2-fermented peach wine, with a high negative score on PC2, was characterized by higher epicatechin, vanillic acid, and catechin. Schp1260-fermented peach wine, with a higher negative score on PC3 and positive score on PC1, was characterized by higher catechin and epicatechin, and lower vanillic acid. The EC1118-fermented sample was characterized by higher procyanidin C1 and syringic acid, cinnamic acid, and chlorogenic acid owing to its high positive scores on PC1 and PC2. Sc1458-fermented peach wine was characterized by higher syringic acid, chlorogenic acid, and cinnamic acid due to its high positive score on PC1. Ck1273- and Hu32337-fermented peach wine had high negative scores on PC1 and PC3, which could be characterized by higher protocatechuic acid, p-coumaric acid, and isorhamnetin. Rm33374 and Pf33372 were characterized by higher protocatechuic acid, p-coumaric acid, and syringic acid owing to their high negative scores on PC1 and positive scores on PC2. The Io31129-fermented sample showed a high negative score on PC1, which could be characterized by higher protocatechuic acid. Sb1465 was characterized by higher chlorogenic acid, syringic acid, vanillic acid, and isorhamnetin owing to its high positive score on PC3.

The yeasts can alter the phenolic composition in fruit wine by producing pectinase and β-glucosidase to facilitate the extraction of phenolics from the fruit matrix and hydrolyze the glycoside phenolic compounds, generating metabolites to form complex phenolic compounds, and adsorbing phenolics with cell wall components [48]. For example, Belda et al. [49] reported that the application of a non-*Saccharomyces* yeast strain with polygalacturonase activity (*Metschnikowia pulcherrima* NS-EM-34) could improve the phenolic extraction during winemaking. Fermentation with a β-glucosidase-producing yeast strain increased the free resveratrol in grape must [50]. The yeasts and their cell wall components have been demonstrated to be able to absorb phenolic compounds in the wine, thereby altering the phenolic composition [37].

However, the metabolism of phenolic compounds and production of microbial enzymes by yeast were very species- and strain-dependent. Zhang et al. [46] noted that a large number of non-*Saccharomyces* yeast strains had better β-glucosidase-producing ability, whereas only some specific *Saccharomyces* strains could produce β-glucosidase, and the β-glucosidase produced by different yeast strains showed different substrate specificity. Polygalacturonase activity was found only in limited non-*Saccharomyces* yeast species [49]. The diversity in metabolic responses and the enzyme production system during fermentation with different yeast strains might account for the differences in phenolic composition among the peach wines fermented by different yeast strains. It has been reported that the phenolic profiles of albino bilberry wines depended primarily on the employed yeasts [51]. The variations in phenolic profiles were also observed in white grape wine, mixed blueberry and grape juice, and alcoholic black currant beverages fermented by different yeast strains [52,53,54,55]. These results are in agreement with the present findings.

Generally, the enzymatic or chemical oxidation of phenolic compounds during fermentation and storage may cause a decline in phenolic content and browning in the wine. The increase in phenolic content in the wine might lead to a higher degree of browning during storage owing to the rich substrates. However, some yeast strains may produce antioxidants such as GSH that can block the oxidation process or consume oxygen to prevent the oxidation as mentioned in Section 3.1.1. The phenolic content in the wine may be influenced by both the promoting effect on phenolic extraction and protecting effect against oxidation. As indicated in Table 1, Ck1273-, EC1118-, Sc2-, Rm33374-, Io31129-, Pf33372-, and Hu32337-fermented peach wines showed less browning, and their phenolic contents were also relatively higher as compared with the Sc1796-fermented sample. However, the correlation between phenolic content and browning degree was not significant. A similar phenomenon was also observed by Nenadis et al. [56], who reported that no correlation between browning and phenolic content could be found in white wines under accelerated oxidation conditions. This suggests that the impact of yeasts on phenolics as well as browning may be more profound.

To date, knowledge regarding the mechanism involved in the alteration of phenolic composition by yeasts in winemaking, especially from fruits other than grapes, is quite scarce. Further research needs to be performed to elucidate the evolution of phenolic profiles as affected by fermentation with different yeast strains in terms of the specific chemical and textural nature of various fruits.

### 3.3. Volatile Profile

#### 3.3.1. General Composition of VOCs

**Figure 6:**
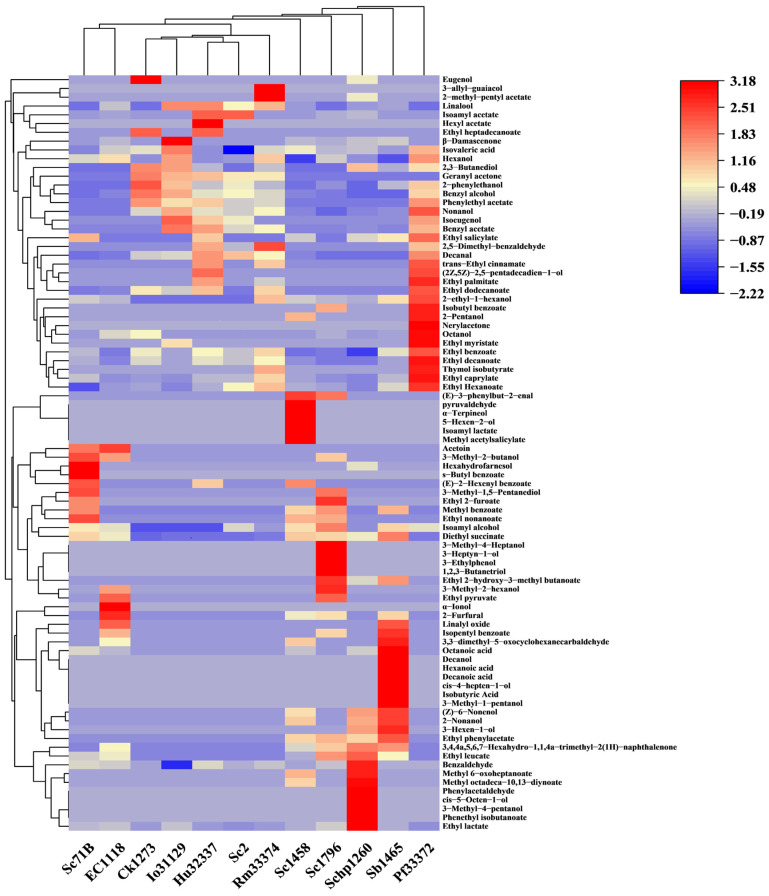
Heatmap of VOCs in peach wines fermented by different yeast strains.

**Figure 7:**
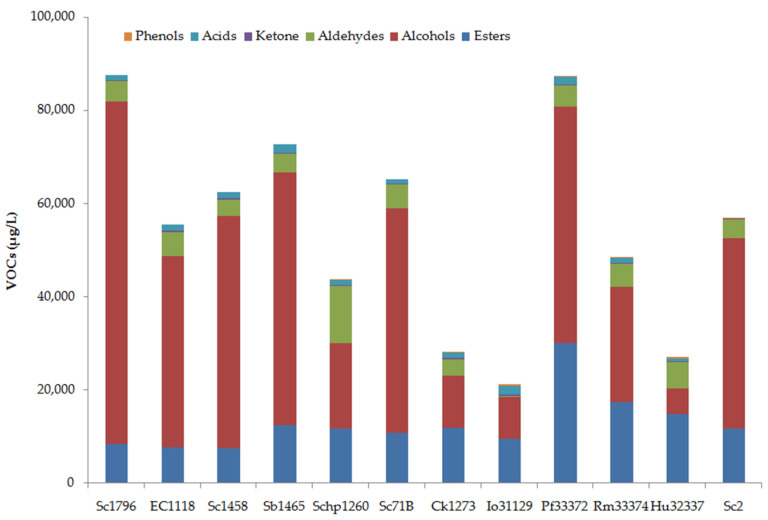
Contents of different groups of VOCs in peach wines fermented by different yeast strains.

By using HS-SPME-GC-MS, a total number of 86 VOCs, including 34 esters, 30 alcohols, 8 aldehydes, 5 ketones, 5 acids, and 4 phenols, were identified in the peach wines fermented by different yeast strains. Figure 6 illustrates the VOCs profiles of the peach wines fermented by different yeast strains, and Figure 7 summarizes the total contents of different groups of VOCs.

As illustrated in Figure 7, esters and alcohols were the most abundant VOCs in peach wines, with contributions ranging from 9.58% to 55.05% for esters and from 19.95% to 83.91% for alcohols, depending on the yeast strain. Notable differences in the volatile profiles could be observed among the peach wines fermented by different yeast strains. The highest content of VOCs was achieved by Sc1796 and Pf33372, with a total amount of 87,567.97 μg/L and 87,361.52 μg/L in the peach wines, respectively. The main VOCs in Sc1796-fermented peach wine were alcohols, contributing to 83.91% of the total VOCs. In the Pf33372-fermented sample, the percentage of alcohols decreased to 58.07%, and the percentage of esters increased to 34.46%. Io31129-, Ck1273-, and Hu32337-fermented peach wines showed the lowest levels of VOCs, being only 24.17–32.29% of that in Sc1796-fermented peach wine. Pf33372 yielded the most abundant esters, with a total amount of 30,103.06 μg/L. Sc1796 produced the highest content of alcohols, while the esters were less.

**Figure 8:**
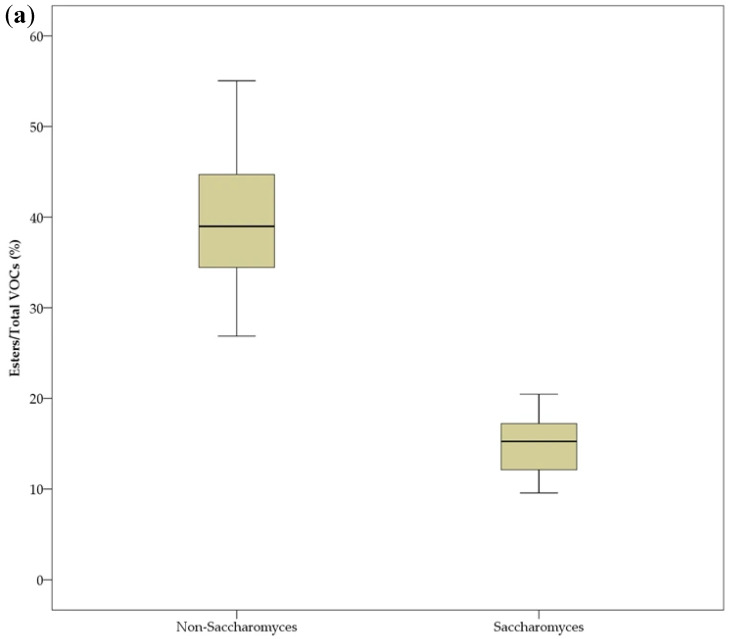

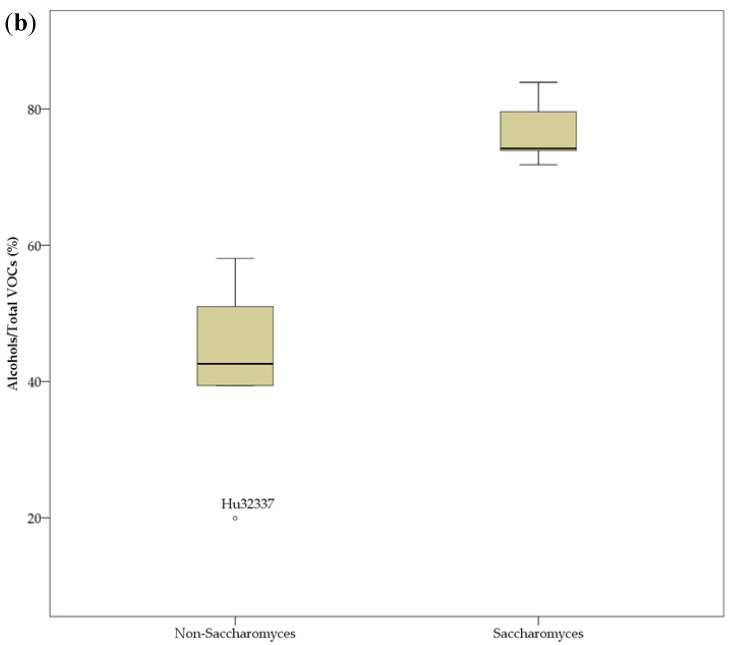
Comparison of percentages of esters (**a**) and alcohols (**b**) in the total content of VOCs of peach wine fermented by *Saccharomyces* and non-*Saccharomyces* yeast strains.

Generally, the non-*Saccharomyces* yeasts tended to produce more esters than alcohols, leading to a high percentage of esters in the total VOCs (Figure 8). Conversely, the *Saccharomyces* strains favored the production of more alcohols than esters, resulting in a higher percentage of alcohols in the total VOCs. There was a significant difference between the two groups of yeasts in terms of the percentages of esters and alcohols in the total VOCs (*p* ˂ 0.05). It has been generally recognized that non-*Saccharomyces* yeast strains have a greater ability to produce volatile esters than *Saccharomyces* strains, and the application of selected non-*Saccharomyces* yeast strains for the improvement of ester production during fermentation has been suggested as a strategy to improve the aromatic profiles of fruit wines [5]. The present results also agree with this. Further studies can be conducted on co-fermentation with selected *Saccharomyces* and non-*Saccharomyces* yeast strains to further improve the quality of peach wine.

#### 3.3.2. Alcohols

Alcohols were the predominant VOCs in most samples except for Hu323337-, Ck1273-, and Io31129-fermented peach wines, in which their levels were exceeded by esters. The alcohols are responsible for the aroma complexity of fruit wine. The main alcohols detected with a higher content and abundance included hexanol, benzyl alcohol, isoamyl alcohol, 2-phenylethanol, and octanol. These compounds may contribute a fruity and floral aroma to the wine [48,57]. Sc1796-fermented peach wine had the highest level of alcohols and isoamyl alcohol. Ck1273-, Io31329-, and Pf33372-fermented peach wines had higher levels of benzyl alcohol and 2-phenylethanol than others. Io31329-, Pf33372-, Rm33374-, and EC1118-fermented samples showed a higher hexanol concentration. Furthermore, Pf33372, Ck1273, and EC1118 produced a higher level of octanol than others. Different yeast strains showed diverse metabolic activities for producing specific alcohols.

Interestingly, several terpenoid and norisprenoid alcohols, including linalool, linalyl oxide, α-terpineol, α-ionol, and hexahydrofarnesol, were detected in some wine samples. Linalool was the most abundant terpene alcohol in the peach wines. It was detected in eight samples, including EC1118-, Sc1458-, Sb1465-, Schp1260-, Io31129-, Rm33374-, Hu32337-, and Sc2-fermented peach wines. The highest level of linalool was observed in Hu32337- and Io31129-fermented samples. The α-ionol, α-terpineol, and hexahydrofarnesol were only detected in EC1118-, Sc1458-, and Schp1260-fermented samples, respectively.

#### 3.3.3. Esters

The major esters detected in the peach wine included ethyl lactate, ethyl caprylate, ethyl decanoate, and ethyl benzoate, which widely existed in most peach wines with higher concentrations. Pf33372-fermented peach wine had the highest level of esters, being 1.73–3.97 times higher than others. Schp1260-fermented peach wine had the highest level of ethyl lactate. Pf33372 showed the strongest ability for producing ethyl caprylate, ethyl decanoate, and ethyl benzoate. Hu32337 and Sc2 produced more isoamyl acetate than others. Phenylethyl acetate was only detected in Ck1273-, Io31129-, Pf33372-, Rm33374-, Hu32337-, and Sc2-fermented peach wines. Ethyl pyruvate was only found in Sc1796- and EC1118-fermented samples. Ethyl leucate was only observed in Sc71B-, Sc1796-, EC1118-, Sc1458-, Sb1465-, and Schp1260-fermented peach wines.

#### 3.3.4. Aldehydes

Schp1260 yielded the highest level of aldehydes in peach wine, accounting for 28.23% of the total amount of VOCs. Benzaldehyde was the predominant aldehyde in peach wine, which could contribute an almond- and cherry-like aroma to the wine [58]. Schp1260-fermented peach wine had the highest level of benzaldehyde. Decanal was another abundant aldehyde in peach wine, existing in eight samples, with Pf33372 and Hu32337 showing higher levels than others.

#### 3.3.5. Ketones

Five ketones, including acetoin, β-damascenone, geranyl acetone, nerylacetone, and 3,4,4a,5,6,7-Hexahydro-1,1,4a-trimethyl-2(1H)-naphthalenone, were detected in the peach wines, but only some specific samples contained them. β-damascenone were detected in six samples, among which Io31129-fermented peach wine showed the highest level with 7.27–17.67 times higher than others. Geranyl acetone was observed in five samples, with Ck1273 showing a higher level than others. Nerylacetone was only found in Pf33372-fermented peach wine.

#### 3.3.6. Acids and Phenols

The most abundant volatile acid in the peach wines was isovaleric acid, existing in 11 samples with a concentration range of 773.7–2024.4 μg/L. Other volatile acids, including isobutyric acid, hexanoic acid, octanoic acid, and decanoic acid, were only detected in limited samples with low concentration. Considering the high odor threshold of volatile acids and their relatively low concentrations in the peach wines, they might make a limited contribution to the wine aroma.

The volatile phenols in the peach wines were rather low, and only could be detected in seven samples. However, some detected phenols, such as eugenol and isoeugenol, have a very low odor threshold, allowing them to contribute a balsamic, clove, herbaceous, honey, and spice aroma to the wine at a low concentration [59]. The isoeugenol was detected in Io31129-, Pf33372-, Hu32337-, and Sc2-fermented peach wines, with Io31129 being the highest. The eugenol was only found in Ck1273- and Schp1260-fermented peach wines. The 3-allyl-guaiacol and 3-ethylphenol were only observed in Rm33374- and Sc1796-fermented peach wines, respectively.

The yeasts can produce several extracellular enzymes, such as esterase, alcohol acetyltransferase, alcohol dehydrogenase, glycosidase, phenol reductase, and decarboxylase, which are responsible for the formation of esters, alcohols, terpenoids, volatile phenols, and acids [12]. However, the abundance of these enzymes is very species- and strain-dependent, leading to diverse influences on the production of specific aroma compounds during fermentation with different yeast strains. It has been suggested that non-*Saccharomyces* yeast strains could produce a wider range of volatile compounds than *Saccharomyces* strains, probably owing to their higher abundance of extracellular enzymes formed during fermentation [5,12,60]. The present results also confirmed the diversity of volatile profiles of peach wines fermented by different yeast strains. The peach wines fermented by non-*Saccharomyces* yeast strains showed more complex and distinct volatile profiles than those of *Saccharomyces* strains.

#### 3.3.7. PCA Analysis

**Figure 9:**
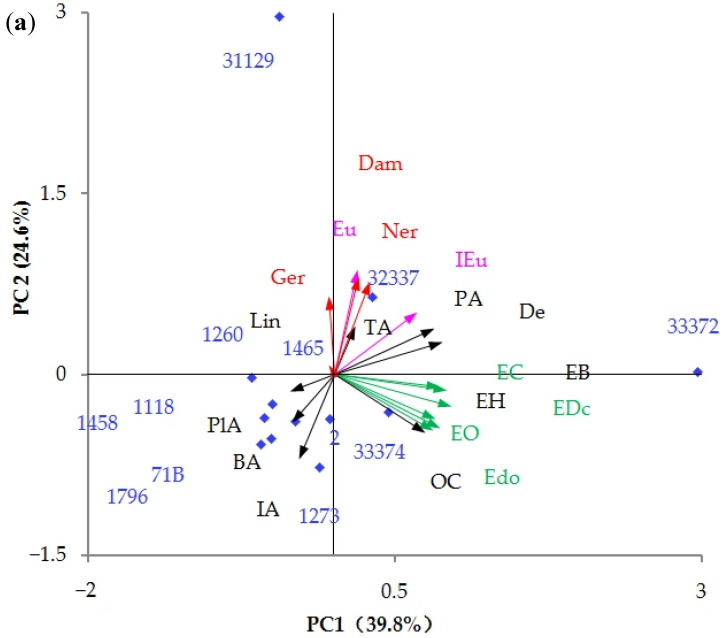

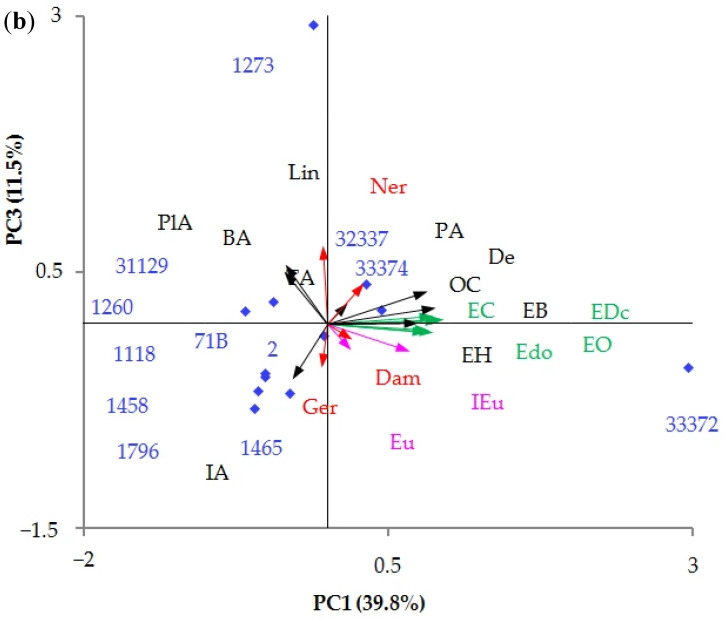

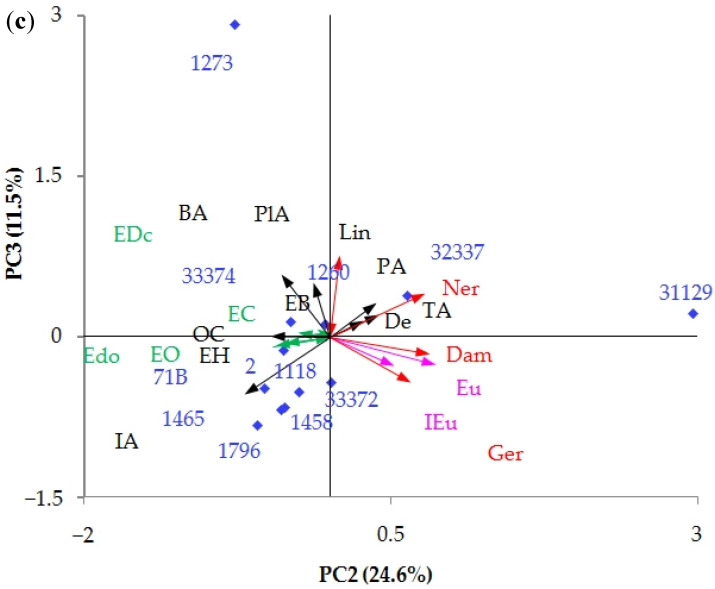
PCA score plots of VOCs with ROAV > 1. (**a**) PC1 vs. PC2; (**b**) PC1 vs. PC3; and (**c**) PC2 vs. PC3. EH: ethyl hexanoate; EO: ethyl octanoate; EDc: ethyl decanoate; Edo: ethyl dodecanoate; EB: ethyl benzoate; EC: (E)-ethyl cinnamate; TA: isoamyl acetate; PA: phenethyl acetate; IA: isoamyl alcohol; Oc: octanol; De: decanal; BA: benzaldehyde; PlA: phenylacetaldehyde; IEu: isoeugenol; Eu: eugenol; Lin: linalool; Dam: β-damascenone; Ger: geranylacetone; Ner: nerylacetone.

To better interpret the influence of different yeast strains on the aroma characteristics of peach wine, the relative odor activity value (ROAV) of each VOC was calculated according to the available odor thresholds in wines from previous studies [57,58,59,61,62]. The data on 19 VOCs with an ROAV > 1, including ethyl hexanoate, ethyl octanoate, ethyl decanoate, ethyl dodecanoate, ethyl benzoate, (E)-ethyl cinnamate, isoamyl acetate, phenethyl acetate, isoamyl alcohol, octanol, decanal, benzaldehyde, phenylacetaldehyde, eugenol, isoeugenol, linalool, β-damascenone, geranylacetone, and nerylacetone, were visualized by using a PCA map. Three principal components (PCs), which could explain 75.87% of the variance in the original data, were extracted for score plots. The ethyl esters, including ethyl hexanoate, ethyl octanoate, ethyl decanoate, ethyl dodecanoate, ethyl benzoate, and (E)-ethyl cinnamate, which mainly contribute to the fruity aroma, were mainly positively loaded on PC1. The eugenol, isoeugenol, β-damascenone, geranylacetone, and erylacetone, which mainly contribute to floral aroma, were mainly positively loaded on PC2. The linalool was mainly positively loaded on PC3. As shown in Figure 9, Pf33372-fermented peach wine had a high positive score on PC1, suggesting it could be characterized by a fruity aroma. However, its floral aroma was faint due to its low positive score on PC2. Conversely, Io31129-fermented peach wine was characterized by a floral aroma due to its high positive score on PC2. The Hu32337-fermented sample was characterized by a moderate fruity and floral aroma owing to its moderate positive scores on PC1 and PC2. Rm33374 was characterized by a moderate fruity aroma owing to its moderate positive score on PC1. SC1796-, EC1118-, Sc1458-, Sc71B-, Schp1260-, Io31129-, and Sb1465-fermented peach wines were characterized by higher phenethyl acetate, isoamyl alcohol, and benzaldehyde, owing to their high negative scores on PC1. These three VOCs can provide the peach wines with floral, fruity and floral, and almond and cherry aromas, respectively [52,53,55].

## 4. Conclusions

Screening for yeast strains with better fermentation performance and improved nutritional and volatile profiles is an essential work for high-quality fruit wine production. In this study, a total number of 12 yeast strains, including five *S*. *cerevisiae* strains, one *S. bayanus* strain, and six non-*Saccharomyces* yeast strains were evaluated for their performance in fermenting fresh-squeezed white-fleshed peach puree into wine. It was found that all of the yeast strains could generally finish the alcohol fermentation, producing ethanol levels within the typical range for fruit wines. The non-*Saccharomyces* yeast strains generally showed lower ethanol production abilities than *Saccharomyces* strains. The peach wines fermented by non-*Saccharomyces* yeasts had higher sugar-free extract and acidity. Different yeast strains showed diverse metabolic activities for accumulating specific phenolic compounds and VOCs. This resulted in different characteristics of phenolic and volatile profiles in peach wines.

The *S*. *cerevisiae* strain EC1118 had the best ethanol production ability. It also yielded the highest TPC, procyanidin C1, and antioxidant capacity, and better retained the natural color of white-fleshed peach pulp. These characteristics suggest that *S. cerevisiae* strain EC1118 may be a suitable candidate for peach wine fermentation. *S. cerevisiae* 1458, *S. bayanus* 1465, and *R. mucilaginosa* 33,374 also showed comparable fermentation ability to EC1118, and they produced lower higher alcohols. *S*. *cerevisiae* strain 2 produced the highest level of epicatechin and vanillic acid. The non-*Saccharomyces* yeast strains *R. mucilaginosa* 33,374, *H. uvarum* 32,337, and *I. orientalis* 31,129 accumulated higher levels of protocatechuic acid.

Esters and alcohols were the most abundant VOCs in peach wines. The non-*Saccharomyces* yeasts produced more esters than alcohols, while the *Saccharomyces* strains produced more alcohols than esters. *P. fermentans* 33,372-fermented peach wine was characterized by a high level of ethyl esters. *I. orientalis* 31,129 produced more isoengenol, linalool, and β-damascenone. The non-*Saccharomyces* yeast strains were more suitable for use individually or in co-fermentation with *S*. *cerevisiae* strains to produce peach wines with a higher level of VOCs.

These findings would help to select suitable yeast strains for producing peach wines with high quality, especially fermentation with fresh-squeezed peach puree or juice prepared from white-fleshed peaches.

## Figures and Tables

**Table 1:** Color parameters of peach wines fermented by different yeast strains.

Yeasts	L*	a*	b*	h*(_°_)	C*
Sc1796	19.29 ± 2.17 ^a^	−0.06 ± 0.21 ^bc^	12.68 ± 0.01 ^a^	89.31 ± 0.54 ^a^	12.68 ± 0.01 ^a^
EC1118	19.53 ± 0.69 ^a^	−0.34 ± 0.34 ^cdef^	8.67 ± 1.20 ^cde^	87.73 ± 1.75 ^bcd^	8.68 ± 1.21 ^cde^
Sc1458	19.17 ± 0.90 ^a^	−0.01 ± 0.20 ^b^	10.09 ± 0.37 ^bc^	89.15 ± 0.52 ^ab^	10.09 ± 0.37 ^bc^
Sb1465	18.73 ± 1.10 ^a^	−0.75 ± 0.20 ^h^	11.22 ± 1.65 ^ab^	86.17 ± 1.01 ^ef^	11.25 ± 1.66 ^ab^
Schp1260	18.64 ± 1.69 ^a^	0.51 ± 0.01 ^a^	8.16 ± 1.47 ^de^	86.33 ± 0.6 ^def^	8.17 ± 1.47 ^de^
Sc71B	19.99 ± 0.83 ^a^	−0.15 ± 0.20 ^bcde^	9.38 ± 1.05 ^cd^	88.81 ± 1.14 ^abc^	9.38 ± 1.05 ^cd^
Ck1273	19.99 ± 1.18 ^a^	−0.11 ± 0.01 ^bcd^	7.51 ± 1.04 ^e^	89.12 ± 0.13 ^ab^	7.51 ± 1.04 ^de^
Io31129	18.91 ± 0.05 ^a^	−0.65 ± 0.06 ^gh^	8.69 ± 0.67 ^cde^	85.69 ± 0.05 ^f^	8.71 ± 0.67 ^cde^
Pf33372	19.83 ± 0.50 ^a^	−0.41 ± 0.06 ^efg^	8.58 ± 0.76 ^cde^	87.21 ± 0.65 ^de^	8.59 ± 0.76 ^cde^
Rm33374	20.16 ± 0.68 ^a^	−0.38 ± 0.18 ^defg^	8.65 ± 0.94 ^cde^	87.47 ± 1.26 ^cde^	8.66 ± 0.94 ^cde^
Hu32337	19.47 ± 2.34 ^a^	−0.57 ± 0.1 ^fgh^	9.07 ± 0.12 ^cde^	86.4 ± 0.67 ^def^	9.08 ± 0.12 ^cde^
Sc2	19.60 ± 0.43 ^a^	−0.57 ± 0.11 ^fgh^	8.38 ± 0.88 ^de^	86.14 ± 0.49 ^ef^	8.40 ± 0.89 ^de^

Values represent the mean ± standard deviation (*n* = 3). L*: Lightness; a*: green-redness; b*: blue-yellowness; C*: chroma; h*: hue angle. Different letters in the same column indicate statistically significant differences (*p* ˂ 0.05).

**Table 2:** General chemical composition of peach wines fermented by different yeast strains.

Yeasts	Ethanol (%, *v*/*v*)	Total Acidity (g/L)	Sugar-Free Extract (g/L)	Residual Sugar (g/L)
Sc1796	11.80 ± 0.26 ^ab^	6.25 ± 0.14 ^c^	26.81 ± 2.65 ^cde^	5.32 ± 0.29 ^de^
EC1118	12.43 ± 0.21 ^a^	4.39 ± 0.27 ^e^	22.73 ± 1.50 ^e^	4.97 ± 1.34 ^de^
Sc1458	11.97 ± 0.49 ^ab^	4.82 ± 0.24 ^de^	21.87 ± 2.92 ^e^	4.80 ± 0.54 ^de^
Sb1465	12.33 ± 0.12 ^a^	5.03 ± 1.32 ^cde^	22.91 ± 2.68 ^de^	3.75 ± 2.12 ^e^
Schp1260	11.13 ± 0.06 ^bcd^	10.89 ± 0.65 ^a^	32.19 ± 1.82 ^abc^	8.54 ± 0.93 ^ab^
Sc71B	11.27 ± 1.15 ^bcd^	6.74 ± 1.87 ^bc^	28.38 ± 4.8 ^bcde^	4.64 ± 0.25 ^de^
Ck1273	10.50 ± 1.04 ^d^	6.09 ± 0.61 ^cd^	34.64 ± 6.96 ^ab^	7.66 ± 1.59 ^bc^
Io31129	11.03 ± 0.96 ^bcd^	7.08 ± 0.21 ^bc^	38.61 ± 8.94 ^a^	6.39 ± 0.27 ^cd^
Pf33372	11.30 ± 0.36 ^bcd^	5.75 ± 1.30 ^cd^	30.42 ± 3.10 ^bcd^	5.08 ± 1.52 ^de^
Rm33374	11.60 ± 0.30 ^abc^	6.02 ± 0.72 ^cd^	31.22 ± 4.55 ^abc^	5.81 ± 0.31 ^cde^
Hu32337	10.80 ± 0.53 ^cd^	8.08 ± 0.49 ^b^	34.76 ± 5.83 ^ab^	10.31 ± 0.78 ^a^
Sc2	11.97 ± 0.06 ^ab^	4.34 ± 0.04 ^e^	20.80 ± 0.82 ^e^	5.46 ± 0.62 ^de^

Values represent the mean ± standard deviation. Different letters in the same column indicate statistically significant differences (*p* ˂ 0.05).

**Table 3:** Phenolic composition of peach wines fermented by different yeast strains (mg/L).

Yeasts	Protocatechuic Acid	Catechin	Vanillic Acid	Chlorogenic Acid	Syringic Acid	Epicatechin	Procyanidin C1	p-Coumaric Acid	Cinnamic Acid	Isorhamnetin
Sc1796	2.52 ± 0.12 ^ef^	3.13 ± 0.04 ^bcd^	1.55 ± 0.78 ^ef^	6.21 ± 0.29 ^ab^	5.27 ± 0.4 ^abcd^	23.75 ± 0.5 ^bc^	2.88 ± 0.11 ^bcd^	0.45 ± 0.06 ^bcd^	0.05 ± 0.02 ^cd^	0.64 ± 0.05 ^cd^
EC1118	2.44 ± 0.11 ^f^	3.19 ± 0.15 ^bc^	2.11 ± 0.04 ^bcd^	6.17 ± 0.21 ^ab^	6.05 ± 1.29 ^ab^	24.45 ± 0.9 ^bc^	5.04 ± 0.79 ^a^	0.44 ± 0.05 ^cd^	0.09 ± 0.01 ^a^	0.78 ± 0.04 ^c^
Sc1458	2.97 ± 0.38 ^ef^	3.67 ± 0.12 ^a^	2.29 ± 0.13 ^b^	6.60 ± 0.37 ^a^	6.27 ± 0.79 ^a^	24.39 ± 3.35 ^bc^	3.09 ± 0.15 ^b^	0.42 ± 0.07 ^cde^	0.08 ± 0.01 ^b^	0.81 ± 0.12 ^c^
Sb 1465	2.42 ± 0.10 ^f^	3.21 ± 0.11 ^bc^	2.23 ± 0.08 ^bc^	6.23 ± 0.05 ^ab^	5.78 ± 0.1 ^abc^	24.64 ± 0.11 ^b^	2.92 ± 0.01 ^bc^	0.39 ± 0.00 ^def^	0.07 ± 0.00 ^b^	1.22 ± 0.01 ^ab^
Schp1260	3.48 ± 0.22 ^e^	3.51 ± 0.07 ^a^	1.09 ± 0.41 ^f^	5.56 ± 0.08 ^c^	4.61 ± 0.2 ^d^	24.01 ± 0.28 ^bc^	2.93 ± 0.06 ^bc^	0.30 ± 0.01 ^g^	0.07 ± 0.00 ^b^	0.76 ± 0.13 ^cd^
Sc71B	2.09 ± 0.06 ^f^	3.39 ± 0.41 ^ab^	1.99 ± 0.26 ^bcde^	6.17 ± 0.33 ^b^	5.58 ± 1.26 ^abcd^	24.49 ± 0.51 ^bc^	2.56 ± 0.22 ^cde^	0.33 ± 0.03 ^fg^	0.06 ± 0.02 ^bc^	0.60 ± 0.06 ^d^
Ck1273	6.68 ± 0.18 ^c^	2.87 ± 0.05 ^d^	1.72 ± 0.22 ^de^	5.52 ± 0.23 ^c^	5.65 ± 0.35 ^abcd^	23.37 ± 0.75 ^bc^	2.46 ± 0.02 ^e^	0.53 ± 0.05 ^ab^	0.04 ± 0.00 ^d^	1.29 ± 0.09 ^a^
Io31129	8.41 ± 0.18 ^ab^	3.12 ± 0.2 ^bcd^	1.68 ± 0.08 ^de^	5.39 ± 0.27 ^c^	5.15 ± 0.04 ^bcd^	23.31 ± 0.22 ^bc^	2.45 ± 0.01 ^e^	0.49 ± 0.01 ^abc^	0.04 ± 0.00 ^d^	1.11 ± 0.03 ^b^
Pf33372	4.61 ± 0.03 ^d^	2.87 ± 0.08 ^d^	1.64 ± 0.1 ^de^	6.46 ± 0.43 ^ab^	5.77 ± 0.31 ^abc^	23.19 ± 0.95 ^bc^	2.50 ± 0.07 ^de^	0.47 ± 0.10 ^bcd^	0.04 ± 0.00 ^d^	1.18 ± 0.01 ^ab^
Rm33374	8.64 ± 0.07 ^a^	3.02 ± 0.09 ^cd^	1.81 ± 0.04 ^cde^	6.58 ± 0.02 ^ab^	5.85 ± 0.32 ^abc^	22.61 ± 0.35 ^c^	2.45 ± 0.01 ^e^	0.56 ± 0.04 ^a^	0.04 ± 0.00 ^d^	1.07 ± 0.09 ^b^
Hu32337	7.61 ± 2.01 ^bc^	3.05 ± 0.04 ^cd^	1.61 ± 0.04 ^e^	5.68 ± 0.25 ^c^	5.21 ± 0.21 ^bcd^	22.85 ± 1.18 ^bc^	2.48 ± 0.03 ^de^	0.46 ± 0.01 ^bcd^	0.04 ± 0.00 ^d^	1.07 ± 0.05 ^b^
Sc2	2.65 ± 0.1 ^ef^	3.39 ± 0.3 ^ab^	4.09 ± 0.07 ^a^	5.62 ± 0.16 ^c^	4.87 ± 0.46 ^cd^	32.67 ± 0.58 ^a^	2.50 ± 0.04 ^de^	0.35 ± 0.01 ^efg^	0.02 ± 0.01 ^e^	1.31 ± 0.24 ^a^

Values represent the mean ± standard deviation. Different letters in the same column indicate statistically significant differences (*p* ˂ 0.05).

## Data Availability

The original contributions presented in this study are included in the article. Further inquiries can be directed to the corresponding author.
